# Gene expression and splicing alterations analyzed by high throughput RNA sequencing of chronic lymphocytic leukemia specimens

**DOI:** 10.1186/s12885-015-1708-9

**Published:** 2015-10-16

**Authors:** Wei Liao, Gwen Jordaan, Phillipp Nham, Ryan T. Phan, Matteo Pelegrini, Sanjai Sharma

**Affiliations:** 1 Division of Hematology-Oncology, UCLA-VA Greater Los Angeles Healthcare System, Los Angeles, CA USA; 2 Department of Pathology, VA Greater Los Angeles Healthcare System, Los Angeles, CA USA; 3 Department of Molecular, Cell and Developmental Biology, UCLA, Los Angeles, CA USA; 4 UCLA West Los Angeles VA Medical Center, 11301 Wilshire Blvd, Bldg 304, Rm E1-115, Los Angeles, CA 90073 USA

**Keywords:** CLL, RNA-sequencing, Differential gene expression, Alternative splicing

## Abstract

**Background:**

To determine differentially expressed and spliced RNA transcripts in chronic lymphocytic leukemia specimens a high throughput RNA-sequencing (HTS RNA-seq) analysis was performed.

**Methods:**

Ten CLL specimens and five normal peripheral blood CD19+ B cells were analyzed by HTS RNA-seq. The library preparation was performed with Illumina TrueSeq RNA kit and analyzed by Illumina HiSeq 2000 sequencing system.

**Results:**

An average of 48.5 million reads for B cells, and 50.6 million reads for CLL specimens were obtained with 10396 and 10448 assembled transcripts for normal B cells and primary CLL specimens respectively. With the Cuffdiff analysis, 2091 differentially expressed genes (DEG) between B cells and CLL specimens based on FPKM (fragments per kilobase of transcript per million reads and false discovery rate, FDR q < 0.05, fold change >2) were identified. Expression of selected DEGs (*n* = 32) with up regulated and down regulated expression in CLL from RNA-seq data were also analyzed by qRT-PCR in a test cohort of CLL specimens. Even though there was a variation in fold expression of DEG genes between RNA-seq and qRT-PCR; more than 90 % of analyzed genes were validated by qRT-PCR analysis. Analysis of RNA-seq data for splicing alterations in CLL and B cells was performed by Multivariate Analysis of Transcript Splicing (MATS analysis). Skipped exon was the most frequent splicing alteration in CLL specimens with 128 significant events (*P*-value <0.05, minimum inclusion level difference >0.1).

**Conclusion:**

The RNA-seq analysis of CLL specimens identifies novel DEG and alternatively spliced genes that are potential prognostic markers and therapeutic targets. High level of validation by qRT-PCR for a number of DEG genes supports the accuracy of this analysis. Global comparison of transcriptomes of B cells, IGVH non-mutated CLL (U-CLL) and mutated CLL specimens (M-CLL) with multidimensional scaling analysis was able to segregate CLL and B cell transcriptomes but the M-CLL and U-CLL transcriptomes were indistinguishable. The analysis of HTS RNA-seq data to identify alternative splicing events and other genetic abnormalities specific to CLL is an added advantage of RNA-seq that is not feasible with other genome wide analysis.

**Electronic supplementary material:**

The online version of this article (doi:10.1186/s12885-015-1708-9) contains supplementary material, which is available to authorized users.

## Background

Chronic lymphocytic leukemia (CLL) is a common leukemia characterized by accumulation of B cells in the blood, marrow and lymphatic tissues. The clinical course is highly variable with biological and genetic heterogeneity in leukemic specimens. A number of genetic alterations have been correlated with prognosis [1–5]; however, the ability to prognosticate outcomes and tailor treatment based on genetic alterations is still limited. To identify genetic alterations in CLL, a number of different methods have been employed including cytogenetic studies [6], and array comparative genomic hybridization CGH [7, 8] and recently whole exome sequencing [9]. The whole exome sequencing of CLL specimens has also resulted in the identification of novel recurring mutations in the *MYD88, NOTCH1, KLH6* and *SF3B1* genes [10].

To study the complete transcriptome of cells, microarrays have been extensively used, and these studies have identified a number of differentially expressed genes [11–14]. Microarray techniques are, however, subject to a number of limitations including, cross hybridization of transcripts, limitation in coverage, inability to resolve novel transcripts and a falsely higher estimation of low abundance transcripts [15–18]. With the development of massive parallel RNA sequencing (RNA-seq) technology, there have been a growing number of genome-wide studies that have analyzed the complete transcriptome cells in different malignancies [18–22] and non-malignant diseases [23, 24]. Besides analyzing the expression level of genes the RNA-seq technology has the added advantage of analyzing expression at the exon level and provides detailed information about alternative splicing variations, novel transcripts, fusion genes, differential transcription start sites and genomic mutations [25, 26]. As all the RNA transcripts are being directly sequenced, this technology is ideally suited to study altered splicing pattern which is especially relevant in cancer cells as they are known to express unique RNA isoforms with varied biological effects [27, 28].

In this study, we performed RNA-seq analysis on CLL specimens and normal peripheral blood B cells to determine transcriptome differences and splicing variations. The data obtained from the RNA-seq analysis was validated by real time PCR on the RNA-seq cohort and a test cohort of specimens. Besides expression analysis a number of novel differentially spliced genes were also identified and analyzed. These findings will facilitate the identification of novel prognostic markers, therapeutic targets and signaling pathways in CLL.

## Methods

### Sample isolation and characterization

Primary CLL specimens analyzed in this study were obtained from untreated CLL patients after appropriate human subject approval. The human subject study was approved by the ethics committee of the West Los Angeles VA Medical Center and an informed written consent was obtained from all patients. A peripheral blood draw was performed, and peripheral blood lymphocytes (PBLs) were isolated by ficoll gradient. In all the CLL specimens, more than 90 % of isolated cells were CD19+ by flow cytometry analysis. Total RNA from isolated B cells (five different normal donors, caucasian males) was purchased from ALLCELLS (Alameda, CA). IGVH mutation (Immunoglobulin variable region heavy chain) analysis was performed on the CLL specimens with multiplexed PCR reactions to assess clonality as previously described [29]. Percentage of CLL cells expressing CD38 marker and Zap-70 (intracellular staining) was determined by flow cytometry and specimens with more than 20 % cells expressing Zap-70 were defined as Zap-70 positive. CLL specimens in a separate test cohort (*n* = 47) were from all clinical stages, chemotherapy naïve, and with more than 90 % CD19+ cells.

### RNA-seq and library preparation

For library preparation, the Illumina TruSeq RNA sample Prep Kit v2 (San Diego, CA) was used according to the manufacturer’s protocol. Briefly, 1 μg of total mRNAs from five normal B and ten CLL cells was poly-A purified, fragmented, and first-strand cDNA reverse transcribed using random primers. Following second-strand cDNA synthesis, end repair, addition of a single A base, adaptor ligation, and PCR amplification, the enriched cDNA libraries were sequenced using the Illumina HiSeq 2000 at the UCLA Broad Stem Cell Research Center High Throughput Sequencing Core. The RNA sequencing data is deposited at GEO website, accession number GSE70830.

### Primary processing and mapping of RNA-seq reads

50 bp single-end RNA-seq reads were obtained from Illumina HiSeq 2000. Sequence files were generated in FASTQ format (sequence read plus quality information in Phred format). RNA-seq data were analyzed using the UCLA Galaxy server (galaxy.hoffman2.idre.ucla.edu). The quality score of RNA-seq reads was obtained by using the FastQC and the mean quality of each base pair in the samples was 28, indicating a good-quality call in the 50 bp reads [30]. Reads were then processed and aligned to the UCSC H. sapiens reference genome (build hg19) using TopHat v1.3.3 [31–33].

### Assembly of transcripts and differential expression

The aligned read BAM files were assembled into transcripts, their abundance estimated and tests for differential expression processed by Cufflinks v2.0.1 [33]. Cufflinks uses the normalized RNA-seq fragment counts to measure the relative abundances of transcripts. The unit of measurement is fragments per Kilobase of exon per Million fragments mapped (FPKM). Confidence intervals for FPKM estimates were calculated using a Bayesian inference method. After assembly with Cufflinks, the output files were sent to Cuffmerge along with a reference annotation file. To normalize multiple samples for differential expression analysis, we utilized a “geometric” method as described in Anders and Huber [34]. For cross-replicate dispersion estimation, a “pooled” method was used in which each replicated condition is used to build a model, and then these models are averaged to provide a single global model for all conditions in the experiment. The expression testing was done at the level of transcripts and genes and pairwise comparisons of expression between normal and CLL samples. Only the comparisons with “q-value” less than 0.05 and expression fold change greater than two fold in the Cuffdiff output were regarded as showing significant differential expression. Downstream analysis for Cuffdiff output was done using CummeRbund [34].

### RT-PCR validation of RNA-seq results

The differentially expressed genes were validated by Quantitative Real-time Polymerase Chain Reaction (qRT-PCR) using a StepOnePlus™ Real-Time PCR System (Life technologies). cDNA templates from five normal B cell and ten CLL cells were analyzed for expression of *DSP, TRIB2, DUSP1, FOS, JUN, SELPLG, AMICA, MMP9, TYROBP, and LEF1* with taqman probes obtained from Applied Biosystems. The probes selected for these genes provide the best coverage so that the majority of transcripts of the gene are quantified (further information is available on request). To analyze the IGVH subgroups, expression of three genes *T, TFEC* and *IGLL5* was also determined with Taqman probes. Expression of a number of reference genes (Actin, Ribosomal protein large PO, phosphoglycerate kinase, Hypoxanthine phoshoribosyl transferase and Transferrin receptor) was tested for expression in CLL and B cells, and actin was selected as the standard reference gene and the data was analyzed by the method of Pfaffll [35].

### Functional annotation of differentially expressed genes

The differentially expressed gene lists were submitted to Ingenuity Pathway Analysis (IPA, Ingenuity Systems). The functional annotation identifies the biological functions that are most significant to the data set. A Fisher’s exact test was used to calculate a *p*-value determining the probability that the association between the genes in the dataset and the functional annotation is explained by chance alone.

### Alternative splicing analysis with MATS

The RNA-Seq data of B cells and CLL specimens was analyzed for splicing alterations. To identify such events, MATS 3.0.8 (Multivariate Analysis of Transcript Splicing, ref [36]) was used to determine junctional reads within ENSEMBL human gene annotations. This software implements a Bayesian approach that detects differential AS (alternative splicing) under two conditions by examining whether the difference in the exon-inclusion levels between two samples exceeds a given user-defined threshold. To identify these events, we used the following criteria, 1. Splicing events were labeled significant if the sum of the reads supporting a specific event exceeded 10 reads, 2. *P*-value was <0.05, and 3. Minimum inclusion level difference as determined by MATS was >0.1 (10 % difference). To validate the splicing alterations RT-PCR analysis was performed by designing primers in the neighboring exons (primer sequences available on request).

## Results

### Analysis of RNA-seq data

**Fig. 1:**
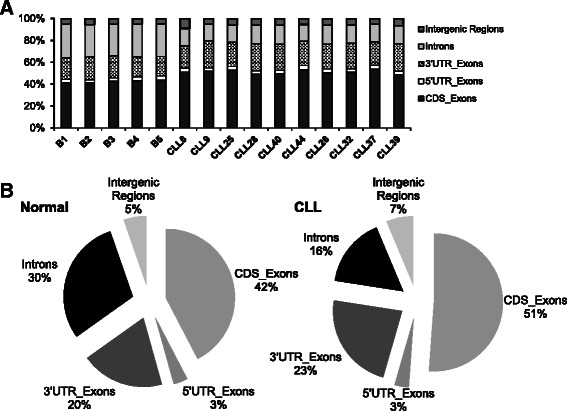
Distribution of sequencing reads in normal B cells and CLL specimens. **a** The bar diagram represents distribution of uniquely mapped reads to human genome UCSC_hg19 (GRCh37). Each bar depicts the percentage of reads from individual samples (five normal B cell and ten CLL specimens) mapped to coding sequence exon (CDS_exon), 5’ and 3’ untranslated regions (5’ and 3’UTR_Exons), introns and intergenic regions. **b** Pie charts represent the average percentage of sequencing reads from five normal B cell (*left*) and ten CLL specimens (*right*) that map to the above mentioned regions

Five normal CD19+ B Cell RNA from different donors (B1 to B5), six IGVH un-mutated primary CLL specimens (CLL6, CLL9, CLL25, CLL28, CLL40, and CLL44) and four IGVH mutated CLL specimens (CLL26, CLL32, CLL37, and CLL39) were subjected to HTS-RNA single-end RNA sequencing (Table 1). The total WBC counts for unmutated IGVH (U-CLL) were higher than mutated IGVH (M-CLL) specimens (Table 1) and the U-CLL specimens were noted to have a higher percentage of leukemic cells expressing CD38 and Zap-70 as described before [4, 5]. The total number of raw reads in B cells (*n* = 5) and CLL (*n* = 10) specimens ranged from 31 to 85 million reads, and 37 to 101 million for normal, CLL, respectively (Fig. 1, Additional file 1). To assess the quality of mapping reads to the reference genome hg19, some key metrics were extracted from the TopHat output, and analyzed using the RNA-seq quality control package RseQC [37]. The majority of reads (between 65.5 % and 79.6 %) are uniquely mapped to the reference genome sequences across all samples (Additional file 1). The mean mapping percentage for normal B cells and CLL specimens is 78.3 % and 74.4 % and 5.8 % to 8.8 % of the reads mapped to the known splice junctions respectively.

**Table 1:** Clinical characteristics of CLL patients and RNA sequencing read count data

	Age sex, Rai stage, total WBC count (cumm^3^)	% cells CD38+	Zap-70 status	Specimen
Normal CD19+ B cells				B1
				B2
				B3
				B4
				B5
U-CLL	67 M, stage II, 40,000	0 %	neg	CLL6
	88 M, stage III, 90,000	59 %	pos	CLL9
	62 M, stage I, 96,000	24 %	pos	CLL25
	71 M, stage II, 135,000	8 %	pos	CLL28
	56 M, stage II, 102,000	55 %	pos	CLL40
	68 M, stage IV, 320,000	50 %	pos	CLL44
M-CLL	55 M, stage III, 37,000	0 %	ND	CLL26
	61 M, stage 0, 24,000	5 %	neg	CLL32
	64 M, stage I, 28,000	0 %	pos	CLL37
	78 M, stage III, 98,000	0 %	neg	CLL39

(neg, negative, pos, positive, ND, not done, M-CLL mutated IGVH, U-CLL non-mutated IGVH)

To further examine the read distribution, the uniquely mapped reads were assigned to: exon coding sequence (CDS_Exons), 5’ and 3’ untranslated regions (UTR_Exons), introns and intergenic regions. In Fig. 1a, the distribution of mapped reads is shown across the samples. 41 % to 52 % of reads mapped to exon coding sequence, 2.9 % to 3.8 % mapped to 5’UTR while 18 % to 25 % mapped to 3’UTR. The introns and intergenic regions account for about 15–30 % and 5–9 %, respectively (data for all specimens is in Additional file 1). To compare if there is a difference in read distribution between normal B cell and CLL, mapping data from Fig. 1a was averaged and plotted as a pie chart in Fig. 1b. The exonic reads (CDS_Exons) were higher in CLL specimens as compared to B cells while intronic reads were higher in the B cell specimens (Fig. 1b), 30 % vs. 16 % for normal B cells and CLL specimens. The high number of reads mapping to introns have been reported in other RNA-seq analysis [38] and could be due to genomic DNA contamination, sequencing of pre-mRNA, novel exons, or nascent transcription and co-transcriptional splicing as described in Ameur et al [39].

### Defining the transcriptomic profiles of normal B cell, and CLL specimens

To examine the transcriptome profile of normal B cells and primary CLL specimens, transcripts were assembled and their expression values calculated using Cufflinks. Pair-wise comparisons of transcriptomic profiles of normal B cells, CLL specimens as well as disease-subtype as determined by IGVH mutational status (U-CLL, un-mutated IGVH and M-CLL, mutated IGVH), were performed. The transcript abundance was calculated by estimating the fragment per kilobase of exon per million mapped fragments (FPKM). The numbers of assembled transcripts for normal B cell, U-CLL and M-CLL were 10396, 10494, and 10402 and the genes identified for the three sample groups were 10081, 10111, and 10068, respectively (Additional file 2A). Overall, the number of transcripts and genes found in three groups are very similar indicating a uniform sequencing depth in the various groups.

To determine significant differences in the transcriptomic profiles in the three sample groups (B, U-CLL and M-CLL), pair wise scatter plots matrix was generated by CummeRbund [34]. This analysis compares and correlates the FPKM profile of all expressed genes in all three sample groups, and it also shows the density distribution of FPKM for genes expressed. In Additional file 2B, the density plot reveals that the FPKM distributions among three sample groups are similar, and the FPKM of all expressed genes ranged from 0.003 to 3000 (log_10_FPKM -2.5 to 3.5), with the majority of the genes expressing FPKM range from 1 to 100 (log_10_FPKM 0 to 2.5). The global profiles of U- and M-CLL show fewer dispersion as compared to plots where normal B cell data is compared to the CLL specimens indicating similar transcriptome profiles of U- and M-CLL specimens.

### Analysis of differentially expressed genes

**Fig. 2:**
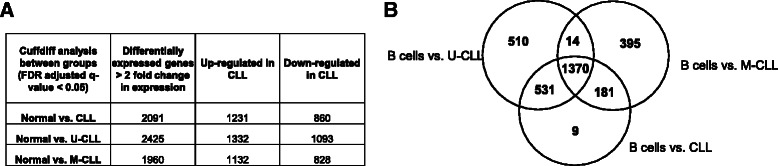
Transcriptomic expression profiles and validation. **a** The number of statistically significant differentially (Up and Down regulated) expressed genes identified from Cuffdiff analysis in various groups relative to B cells are shown in a table format. The differentially expressed genes (DEG, FDR-adjusted q-value < 0.05, Fold change > 2) in all CLL specimens (*n* = 10), U-CLL (*n* = 6) and M-CLL (*n* = 4) was compared to normal B cells. **b** Venn diagram illustrates the overlapped DEG between the three groups in panel A

To determine the differentially expressed genes (DEG) between normal B cells and CLL specimens a Cuffdiff analysis was performed. After filtering differential expressed genes with FDR-adjusted (FDR false discovery rate) q value < 0.05 and fold change > 2, there were 2091 DEG genes between CLL specimens and normal B cells (Fig. 2a). Among these genes, 1231 were up-regulated in CLL and 860 genes were down-regulated (complete gene list in Additional file 3), and the top twenty genes in each group are shown in Table 2. The data was also analyzed by segregating CLL specimens based on their IGVH status and comparing them with normal B cells separately. With this analysis 2425 and 1960 DEG genes were identified in U- and M-CLL specimens respectively. Among these genes, 1332 and 1132 were up-regulated and 1093 and 828 were down-regulated in U-CLL and M-CLL (Fig. 2a). In order to find out if there are overlapping genes that are differentially expressed in both U-CLL and M-CLL samples, the gene lists from normal B cells vs. CLLs, normal B cells vs. U-CLL and normal B cells vs. M-CLL were compared to generate a Venn diagram (Fig. 2b). A high number (1382 genes out of 2091) of differentially expressed genes between normal B cells and CLLs were common to the U-CLL and M-CLL specimens, indicating that this subgroup includes a common set of differentially expressed genes.

**Table 2:** Top twenty up (positive fold change) and down-regulated (negative fold change) genes in CLL versus Normal B cells

Up-regulated genes				Down-regulated genes			
Genes	Description	Fold change	q-value	Genes	Description	Fold change	q-value
FSTL1	follistatin-like 1	1360 ± 4372	0.0090	SYN3	synapsin III	−270	0.0039
MMP9	matrix metallopeptidase 9 (gelatinase B, 92 kDa)	1060 ± 1501	0.0323	DSP	desmoplakin	−179	0.0222
FMOD	fibromodulin	789 ± 1041	0.0008	FBLN2	fibulin 2	−134	0.0074
CXCL5	chemokine (C-X-C motif) ligand 5	593 ± 1008	0.0043	ENAM	enamelin	−117	0.0103
ADTRP	androgen-dependent TFPI-regulating protein	586 ± 485	0.0031	HDC	histidine decarboxylase	−103	0.0270
KSR2	kinase suppressor of ras 2	528 ± 750	0.0232	CD1A	CD1a molecule	−99	0.0311
THBS1	thrombospondin 1	506 ± 640	0.0008	MYO1B	myosin IB	−73	0.0112
TGFBR3	transforming growth factor, beta receptor III	459 ± 205	0.0392	LOC100505738	uncharacterized LOC100505738 or MIR4458	−66	0.0008
CYP1B1	cytochrome P450, family 1, subfamily B, polypeptide 1	423 ± 512	0.0191	SLC45A3	solute carrier family 45, member 3	−47	0.0008
IL8	interleukin 8	411 ± 462	0.0015	MMRN1	multimerin 1	−46	0.0090
CD300E	CD300e molecule	401 ± 650	0.0251	PPFIBP1	PTPRF interacting protein, binding protein 1 (liprin beta 1)	−45	0.0031
PRF1	perforin 1 (pore forming protein)	388 ± 265	0.0106	ZNF618	zinc finger protein 618	−44	0.0015
GIMAP7	GTPase, IMAP family member 7	324 ± 305	0.0323	UACA	uveal autoantigen with coiled-coil domains and ankyrin repeats	−44	0.0083
CTLA4	cytotoxic T-lymphocyte-associated protein 4	276 ± 255	0.0025	AHNAK2	AHNAK nucleoprotein 2	−44	0.0008
CD8A	CD8a molecule	274 ± 257	0.0173	GATA2	GATA binding protein 2	−39	0.0121
NRP1	neuropilin 1	263 ± 475	0.0488	PARM1	prostate androgen-regulated mucin-like protein 1	−39	0.0008
SFTPB	surfactant protein B	261 ± 477	0.0052	CR1	complement component (3b/4b) receptor 1	−39	0.0008
TNFRSF1A	tumor necrosis factor receptor superfamily, member 1A	240 ± 140	0.0052	CABYR	calcium binding tyrosine-(Y)-phosphorylation regulated	−39	0.0264
HBB	hemoglobin, beta	234 ± 553	0.0020	LOC100506178	uncharacterized LOC100506178	−36	0.0209
CYBRD1	cytochrome b reductase 1	216 ± 254	0.0020	FFAR1	free fatty acid receptor 1	−34	0.0224

(q value: adjusted p-value)

To validate the RNA-seq data, a number of differentially expressed genes with potential biological relevance to CLL were selected from this analysis, and their FPKM data was compared to the expression level by real time RT- PCR (qRT-PCR). In an initial experiment the expression level of a number of reference genes in normal B cells and CLL specimens was determined to identify the appropriate reference gene. Actin, Ribosomal protein large PO, Phosphoglycerate kinase, Hypoxanthine phoshoribosyl transferase and Transferrin receptor expression was analyzed with Taqman probes and the expression of actin was the most abundant in all the CLL (*n* = 3) and B cell specimens (*n* = 3) and was selected as the standard reference gene. *FOS* (# 111), *JUN* (#152), *DSP* (desmoplakin #2), *TRIB2* (Tribbles homolog 2, #66) and *DUSP1* (dual specificity phosphatase 1 # 49) were selected from the set of genes that have a lower expression in CLL specimens than B cells (Table 2, Additional file 3, # represent the position of the gene based on the FPKM data, lower number corresponds to higher down regulation). *AMICA1* (#48), *MMP9* (#2), *TYROBP* (#49), *SELPG* (# 604), *LEF1* (#64) were selected as candidate genes to compare the fold over-expression by the two methodologies (# represents fold over-expression relative to B cell based on FPKM data, smaller number indicates higher fold overexpression).

**Fig. 3:**
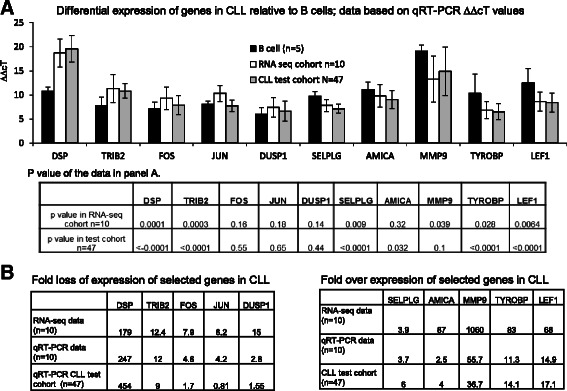
Validation analysis of selected differentially expressed genes. **a** qRT-PCR of selected genes on B cells (*n* = 5), CLL specimens, RNA seq cohort (*n* = 5) and CLL specimens, test cohort (*n* = 47). Data shown is the delta delta cT relative to actin. (Mean and standard deviation). Table below panel A shows the P-values of the qRT-PCR data for the comparison of B cells and CLL RNA seq cohort (*n* = 10), and B cells and CLL test cohort. (*t*-test). **b** Fold expression of selected genes in the larger CLL cohort (*n* = 47) based on qRT-PCR analysis. * PTPRK expression was not detected in normal B cells therefore fold change could not be calculated

The RNA from the identical 10 CLL specimens and five normal B cells (control) was used to perform Taqman probe based qRT-PCR assays. Probes selected for expression analysis provide the best coverage for a particular gene. Figure 3a, b shows three sets of data for each gene expression (*n* = 10), expression based of FPKM values in RNA-seq cohort (*n* = 10), qRT-PCR data from the identical specimens (*n* = 10) as RNA-seq cohort (relative to actin) and qRT-PCR data of a test cohort (*n* = 47, relative to actin) of CLL specimens. Figure 3a bar diagram depicts average ∆∆cT values in the three cohorts with the table below showing the *p* values of data in Fig. 3a. For down regulated genes in CLL, only *DSP* and *TRIB2* expression is significantly lower (*p* < 0.5) as compared to B cells, while in the set of up-regulated genes, the expression of *SELPG, AMICA, TYROBP* and *LEF1* is significantly higher (*p* < 0.5) in the test cohort. *MMP9* expression though significantly higher in the smaller RNA-seq cohort is not significantly higher in the test cohort.

Figure 3b Table compares the fold expression obtained by these analysis, as an example in the case of *DSP* the difference in cT values between actin and *DSP* RNA is around 10 cycles while the expression in CLL cohorts is around 8 cycles lower, i.e. 256 fold fold down regulation of DSP expression in CLL specimens as compared to B cells. This lower *DSP* expression in CLL specimens is similar to the results obtained from FPKM analysis (179 fold lower expression in CLL). *FOS* and *JUN* expression based on RNA-seq FPKM data is 7.9 and 6.2 fold less than B cells while based on the qRT-PCR analysis their expression is 4.6 and 4.2 fold less than B cells. However in the test cohort (*n* = 47) the lower expression of *JUN* cannot be confirmed and for *FOS* the fold lower expression is less than the RNA-seq data (1.7 vs 7.9). Similar variability in expression is observed for *MMP9* and *AMICA1* expression as the fold expression vary 20 to 35 fold when analyzed by RNA-seq FPKM and qRT-PCR. The analysis shows that genes identified as differentially expressed by RNA-seq can be confirmed by qRT-PCR analysis, however the fold expression obtained by the two analysis are variable. Also confirmation with qRT-PCR in additional primary CLL specimens is required as there is significant variability of expression in leukemic cells.

Based on this analysis, additional DEG genes were selected to further compare the two methodologies for RNA expression (Table 3). FPKM and qRT-PCR fold expression levels were compared in the RNA-seq cohort and a test cohort of CLL specimens (*n* = 22). Nine downregulated genes from the RNA-seq data were randomly selected and their expression compared to qRT-PCR analysis. In the case of PTPRK, expression in normal B cells was not identified by qRT-PCR and therefore the RNA-seq data could not be validated. In the case of CCD69, the expression by RNA-seq and qRT-PCR is similar but this lower expression is not observed in our test cohort. Besides these two examples, qRT-PCR confirms a lower expression of these genes in CLL specimens as compared to normal B cells. Twelve genes with a range of over-expression were randomly selected from the list of over-expressed genes from the RNA-seq analysis (Additional file 3) and analyzed by qRT-PCR (Table 3). All the genes were found to be over-expressed based on qRT-PCR in the RNA-seq and test cohort however the expression was variable. Difference in fold expression was observed when the identical specimen was tested by both methodologies as well. Possible explanations for this discrepancy are the normalization of RNA-seq data and the use FPKM for calculation while qRT-PCR analysis is relative expression to a housekeeping gene and the Taqman probe may not provide coverage for all the transcript variants.

**Table 3:** Validation of twenty one differentially expressed genes in CLL. Data from RNA seq analysis (*n* = 10), qRT-PCR of identical specimens (*n* = 10) and qRT-PCR from a test cohort (*n* = 22) of CLL specimens

CLL downregulated genes	Fold downregulation based on RNA-seq data (n =10)	Fold downregulation RNA-seq cohort (qRT-PCR data, n = 10)	Fold downregulation in test cohort qRT-PCR, n = 22)
UACA, uveal autoantigen with coiled-coil domains and ankyrin repeats	44	9.8 ± 3.9	135 ± 3.7
PTPRK, protein tyrosine and phosphatase, receptor type, K	24.2	*	*
JUP, junction plakoglobin	13.4	44 ± 8.1	32 ± 5
ITGA4, integrin, alpha 4 (CD49D, alpha 4 subunit of VLA-4 receptor)	10.3	6 ± 2.9	12.9 ± 2.5
BANK1, B-cell scaffold protein with ankyrin repeats 1	7.2	1.23 ± 2	10.1 ± 1.86
RHOB, ras homolog family member B	5.9	6.5 ± 2	1.97 ± 2.1
Jam3, junctional adhesion molecule 3	5.59	3.5 ± 2.5	75.5 ± 3.6
CD69, CD69 molecule	4.7	4.6 ± 1.7	1.07 ± 2.6 (upregulation)
GRASP, (receptor for phosphoinositides 1)-associated scaffold protein	2.6	36.7 ± 6	9.8 ± 3.8
CLL upregulated genes	Fold downregulation based on RNA-seq data (n =10)	Fold downregulation RNA-seq cohort (qRT-PCR data, n = 10)	Fold downregulation in test cohort qRT-PCR, n = 22)
THBS1, thrombospondin 1	528 ± 640	9.8 ± 15.8	12.9 ± 30.8
TGFBR3, transforming growth factor, beta receptor III	373 ± 205	6.6 ± 1.6	30.5 ± 42
GIMAP7, GTPase, IMAP family member 7	340 ± 305	34.2 ± 3.4	9.7 ± 7.7
LYZ, lysozyme	41.8 ± 69	1.5 ± 5.6	10 ± 9.9
PDE4a, phosphodiesterase 4A, cAMP-specific	26.5 ± 33.9	3 ± 3	4 ± 2.3
MYL9, myosin, light chain 9, regulatory	24.9 ± 34	3.5 ± 3.4	8.7 ± 8.9
RAPGEF 3, Rap guanine nucleotide exchange factor (GEF) 3	24 ± 14.7	17.1 ± 2.9	13.6 ± 11.3
PIM1, pim-1 oncogene	13.9 ± 17	9.4 ± 7.9	6.75 ± 4.6
RXRA, retinoid X receptor, alpha	13.2 ± 12.8	215 ± 196	9.4 ± 9
LCK, lymphocyte specific protein tyrosine kinase	4 ± 2.4	3.2 ± 2.68	4.59 ± 2
CLNK, cytokine-dependent hematopoietic cell linker	3.3 ± 1.8	26 ± 22	36.8 ± 45.2
TGFbeta 1, transforming growth factor, beta 1	1.6 ± 1.1	1.07 ± 2.8	1.4 ± 2.7

*PTPRK expression was not detected in normal B cells therefore fold change could not be calculated

**downregulation of CD69 not validated in test cohort

### Functional pathway analysis

The functional analysis tool was used to categorize genes that were differentially expressed in CLL specimens. Genes from Additional file 3 were analyzed by IPA analysis. The output of the functional annotation is shown in Additional file 4 and the list of genes in each pathway are in Additional file 5. The highest number of DEG genes are in the cell death and survival group correlating well with the unique biological characteristic of CLL, namely resistance to apoptosis. Other significant clustering of genes is observed in cellular movement, cellular development, cellular growth and proliferation and cancer pathways.

### Comparison the CLL IGVH mutated and non-mutated transcriptomes

**Fig. 4:**
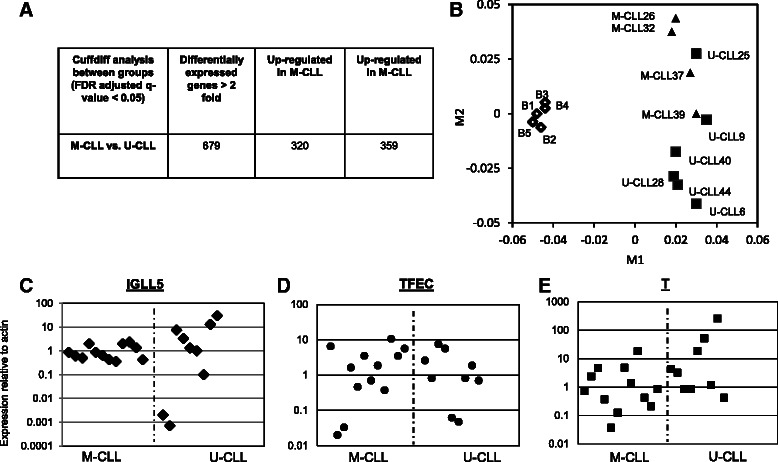
Transcriptomic comparison of IgVH mutated (M-CLL) and non-mutated (U-CLL). **a** Table with Cuffdiff data showing significant differentially expressed genes between M- and U-CLL specimens. **b** MDS plot (Multi-Dimensional Scaling) shows the clustering of the transcriptomic expression profiles of normal B cells (B1-B5), U-CLL and M-CLL samples (numbered as in Table 1). Axes in the MDS plot (M1 and M2) are arbitrary, and the values on the axes are distance units. **c**, **d**, **e** qRT-PCR data from the RNA-seq cohort of CLL specimens (*n* = 10) for three selected genes (*T, IGLL5 and TFEC*) (relative to actin, log scale). These panels show the scatter-plot qRT-PCR data in a separate cohort of CLL specimens and compare the expression of the three selected genes in M and U-CLL specimens. The dotted line separates the M- and U-CLL specimens

Based on the Cuffdiff analysis in Fig. 2a and b a number of genes are differentially expressed in the two CLL subsets, M and U-CLL. A total of 679 genes were more than 2 fold up or down regulated when the average FPKM data of all the genes was compared in the two subsets (Fig. 4a, Additional file 4). To determine whether global transcriptome analysis could segregate the CLL specimens based on IGVH status, a multi-dimensional scaling (MDS) plot (Fig. 4b) was constructed based on their complete transcriptomes. This analysis visualizes the level of similarity of individual samples within a group. MDS analysis was able to segregate the five normal B cells (B1-B5) as they cluster together away from the ten CLL specimens. The CLL specimens, U-CLL (closed boxes) and M-CLL (closed triangles) appear to be separate from each other but there is overlap of CLL specimens #25, #39, #37. Lack of clear separation of specimens on this plot indicates that based on the transcriptome data, M- and U-CLL specimens are not fully distinguishable.

In Additional file 6, the list of differentially expressed between the two groups (U-CLL and M-CLL) is shown that was obtained by dividing the mean FPKMs of the two sub-groups. From this list, we identified three genes for further analysis on an additional cohort of CLL specimens to determine if the expression of these genes is different in these two subgroups. The expression of *IGLL5* (immunoglobulin lambda-like polypeptide 5, immunoglobulin lambda-like polypeptide) and *T* (brachyury homolog, embryonic nuclear transcription factor), was higher in the U-CLL group (top twenty most over-expressed genes in U-CLL as compared to M-CLL) and the expression of *TFEC* (transcription factor EC) was similarly higher in M-CLL group. The expression of these genes was determined by qRT-PCR in a separate cohort of 21 CLL specimens (relative to actin) and is shown in Fig. 4d, e and f scatter plots. The dotted line divides the U- and M-CLL specimens and the expression of these genes in the two sub-groups of CLL specimens is not significantly different when additional CLL specimens are analyzed. The expression of these three specific genes and the transcriptome as a whole for the U- and M-CLL specimens are similar.

### RNA splicing alterations in CLL specimens

**Fig. 5:**
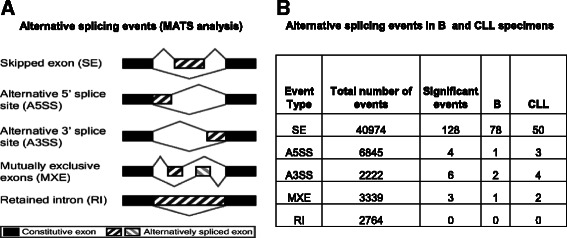
Alternative splicing events in B and CLL specimens. **a** Schematic showing alternative splicing (AS) events (from MATS analysis website). **b** Table with MATS analysis data with different AS events, total events and significant events are shown. B and CLL columns indicate the events out of all the significant events that had higher inclusion levels in either B or CLL specimens

Besides accurately identifying the expression of the genes, the RNA–seq data is also useful in characterizing alternative splicing events. Splicing alterations in CLL specimens can alter the type of transcripts and thereby function of a large number of cellular proteins that may provide the cell with survival advantage. To define the splicing alterations in CLL specimens, the available RNA-seq data was analyzed by MATS (Material and Methods). Fig. 5a is a schematic of the various alternative splicing (AS) events that were analyzed and the number of events identified are listed in Fig. 5b. The analysis identifies AS events both in normal B cells and CLL specimens. Skipped exon (SE) is the most common splicing abnormality with 40974 events of which 128 events passing the threshold for significance. The complete lists of all the significant splice events in Fig. 5b table is in Additional file 7. The significant events in Fig. 5b table are divided into two columns, B and CLL which indicate whether the splicing event led to a higher inclusion of the exon in B or CLL specimens, e.g. 78 SE events resulted in a higher inclusion of exon in B cells and in 50 events, the inclusion of the exon was higher in CLL specimens.

**Fig. 6:**
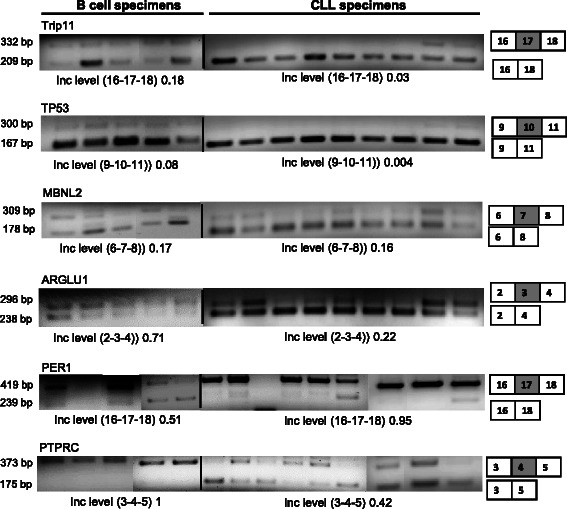
Validation of alternative splicing events. RT-PCR analysis of six AS events. For each gene, five B cell specimens and nine CLL specimen was analyzed. Expected bp (base pair) of the DNA fragments, with schematic of the skipped exon and mean Inc level (inclusion level, based on gel densitometry) are shown

As the SE events, were by far the predominant events they were analyzed by RT-PCR analysis. Sixteen genes (listed in Additional file 8) were selected for initial analysis and primers were designed in the neighboring exons. To confirm DNA amplification of alternatively spliced exons, RT-PCR analysis was performed on B and CLL specimen (Additional file 8). Out of sixteen SE events in two genes there was no PCR amplification and in three genes only one DNA fragment corresponding to a single transcript was amplified (gels in Additional file 8). Low level of transcripts that are not amplified by the PCR is a likely reason that SE events could not be confirmed in these three genes. From the remaining eleven SE events *TRIP11, TP53, MBNL2, ARGLU1, PER1, and PTPRC* genes were randomly selected for further analysis and RT-PCR analysis was performed on B cell (*n* = 5) and CLL specimens (*n* = 9, Fig. 6). For each SE event Fig. 6 describes the exon of the gene that is alternatively spliced, expected base-pair size of the transcript with and without skipped exon along with average Inc. level (inclusion level, based on DNA band densitometry). *TRIP11* (thyroid hormone receptor interactor 11 protein), tumor suppressor p53 (*TP53*), *ARGLU1* (arginine and glutamate rich-1), *Per1* (period 1) and *PTPRC* (CD45) demonstrate at least a two-fold difference in inclusion level of the SE exons in their transcripts. The analysis for *MBNL2* (Muscleblind-like splicing regulator 2) did not show any inclusion level difference between normal B and CLL specimens.

## Discussion

Accurate transcriptome analysis is crucial for determining the expression of genes and thereby activity of signaling pathways that result in growth and survival of leukemic cells. The data from HTS RNA sequencing is an improvement over previous methodologies to effectively and efficiently evaluate the entire transcriptome. The RNA-seq data allows additional analysis of splicing alterations, transcriptional start sites, identification of novel signaling pathways and molecular categorization of specimens that is not feasible with prior genome analytic techniques. With improvement in HTS-sequencing technologies and reduction in the cost of sequencing it is now feasible to compare clinical and biological characteristics of CLL specimens with their global transcriptome profile.

In this study 20 % of genes are identified as differentially expressed (FDR q value < 0.05 and fold change > 2) in CLL specimens as compared to primary B cells. Recently, Ferreira et al has reported RNA-seq and transcriptome analysis of a large cohort of CLL specimens [40]. They report 1089 differentially expressed genes (DEG) between normal B cells and CLL specimens (FDR < 0.01 and median fold change of more than 3). This compares well to our analysis of 2091 DEG genes with a slightly less stringent FDR of < 0.05 and fold change more than 2. A number of DEG genes identified by this analysis were also reported by Ferreira et al [40], e.g. *FOS, JUN, CYBRD1, GZMB, FMOD, CTLA-4,* etc. In this study, data from the RNA-seq analysis was additionally also validated by qRT-PCR in a separate cohort of CLL specimens. Even though a similar expression trend in expression is observed when the genes from the RNA-seq analysis are validated by qRT-PCR, in some instances there can be wide variation based on FPKM and qRT-PCR. There are a number of reasons for the observed differences, e.g. the library preparation for RNA-seq analysis uses mRNA as starting material while total RNA was used for qRT-PCR. Another reason is that the RNA-seq analysis uses the FPKM method for normalization while actin (reference gene) was used as a control with qRT-PCR. Even though the Taqman probes with maximum coverage were used for the assay, but it is possible that some transcript variants were not analyzed by qRT-PCR as we observe that qRT-PCR under-estimates the level of expression as compared to the RNA-seq data in some genes.

Some of the DEG genes identified in this study have been reported earlier in microarray studies, e.g. *MMP-9* and *FMOD* (fibromodulin) over-expression in CLL specimens has been described [41, 42]. *MMP-9*, matrix metallopeptidase 9, functions by degrading a number of matrix proteins such as type IV collagen, the major component of basement membranes. This gene was found to be highly expressed by CLL cells present in the bone marrow and lymph nodes, and contribute to B-CLL progression by facilitating cell migration and tissue invasion. *FMOD*, fibromodulin, is a member of a family of small interstitial proteoglycans and a component of the extracellular matrix that may also regulate TGF-beta activities by sequestering TGF-beta into the extracellular matrix [43]. SiRNA knock-down of this gene results in apoptosis of CLL, indicating its role in CLL survival [42]. *SEPLG, TYROBP*, *LEF1 and AMICA1* genes were significantly up regulated in a number of CLL specimens. *SELPLG* (CD162) is a cell adhesion molecule that is the counter-receptor for selectins and plays a role in lymphocyte trafficking. High expression of *SELPLG* could potentially aid leukemic cells in trans-endothelial migration by interacting with the selectins on the endothelium cells [44]. *TYROBP* or Dap-12 is transmembrane protein that contains ITAM motifs (immunoreceptor tyrosine based activation motif) that are also present in the B-cell receptor (BCR) signaling components [45]. ITAM motifs are central to BCR signaling as a number of signaling molecules and adapter proteins assemble at these motifs. *LEF1* (lymphoid enhancer-binding factor-1) gene encodes a transcription factor that participates in wnt signaling pathway that is active in CLL specimens. *LEF1* is also involved in the transcriptional activation of Myc and CyclinD1, and both these genes are also up regulated in CLL leukemic cells [46]. *AMICA1* expression was marginally higher in the larger cohort of CLL specimens (*p* = 0.032) and is a membrane protein that interacts with CXADR antigen expressed on epithelial and endothelial cells [47]. Table 3 lists additional DEG genes that were confirmed by qRT-PCR analysis. Pim1 kinase over-expression has been reported and this phosphorylates CXCR4 receptor that in turn mediates microenvironment signaling [48]. Similarly PDE4 transcripts in CLL specimens have been described [49] and Lck is associated with B-cell receptor signaling and blocking LCk function results in apoptosis [50].

40–50 % of the DEG genes demonstrate loss of expression in CLL specimens as compared to B cells. *FOS* and *JUN* down regulation in CLL specimens has been reported but in this study, the expression was not significantly lower when this was studied in a larger cohort of specimens [51–53]. One of the mechanisms of *FOS* down regulation is by its interaction with *TCL*1 oncogene that is a potential mechanism of resistance to apoptosis observed in CLL cells. *DSP* (desmoplakin) and *TRIB2* expression were significantly lower in CLL specimens. *DSP* is a key component of the desmosomes that form intercellular junctions and loss of its expression is associated with more invasive behavior of cells [54]. It can also potentially function as a tumor suppressor gene by inhibiting the Wnt/β-catenin signaling pathway [55]. TRIB2 is a member of the Tribbles family of proteins that are similar to serine-threonine kinases but lack catalytic function. These proteins are highly conserved and modulate a number of signaling pathways [56]. *DUSP1,* is a phosphatase that controls cell proliferation and its expression was not significantly lower in the larger cohort of CLL specimens in this analysis [57] as compared to B cells. Additional genes that are downregulated or silenced in CLL specimens are listed in Table 3. UACA (Uveal autoantigen with coiled-coil domains and Ankyrin repeats) that regulates expression of an apoptotic regulator APAF1 [58]. JUP (junctional plakoglobin or gamma-catenin) associates with cytoplasmic domains of cadherins and has tumor and metastasis suppressor activity [59]. Based on their reported functions both these genes are potential tumor suppressor genes in this leukemia as well.

Based on the MDS (multi-dimensional scaling) analysis of the RNA-seq data, the normal B cells and CLL specimens could be segregated on a two-dimensional plot scaling plot. However, the transcriptome data does not clearly distinguish the U- and M-CLL transcriptomes as there is overlap on the scaling plot. The two sub-groups have important biological and clinical differences [60], but their transcriptomes are not significantly different. Expression analysis of selected genes (*T, IGLL5 and TFEC,* ref [61–63]) in the two IGVH sub-groups gave a similar result with no significant difference of expression. The study by Ferreira et al [40] reached an identical conclusion as their analysis could not detect significant transcriptome differences in these two groups. This has also been the observation of other groups that have performed microarray analysis of CLL specimens and have reached a similar conclusion [13, 14].

Alternative splicing events add another layer of complexity besides genes expression as they can alter the structure and function of cellular proteins. Skipped exons are the most common alternative spliced events in CLL specimens in this study and cancer cells in general [27, 28]. Splicing abnormalities have increasingly become more relevant in CLL with the identification of mutations in SF3B1, a splicing factor in a small subset of CLL patients [9] that confer poorer prognosis and can alter RNA splicing patterns. In our analysis, we focused on differentially exon skipping (SE) events as they were by far the most frequent events as compared to alternative 5’, alternative 3’, mutually exclusive exons, and retained intron. Confirmation of different inclusion levels in CD45 (*PTPRC*, a phosphotyrosine phosphatase), *TP53, ARGLU1, PER1,* and *Trip11* genes by RT-PCR indicates that RNA-seq data can be analyzed for splicing alterations. Alternative exon usage in some of these genes such as PTPRC and p53 is well described in previous studies [64–68]. *PTPRC* is a member of the protein tyrosine phosphatase family that has a role in antigen receptor signaling, B cell development and may modulate signaling via integrins and cytokine receptors [66]. A number of studies have characterized expression of *PTPRC* (CD45) isoforms in CLL leukemic cells due to splicing in exons 4, 5 and 6 that alter the extra-cellular domain of the protein. It is however, not well understood whether exon skipping and expression of a particular isoform changes the function of this phosphatase. *Period1* is a gene expressed in a circadian pattern with probable tumor suppressor function and its alternative splice forms though reported have not been characterized [69]. Alternative exon usage in less well characterized genes *Trip11* and *ARGLU1* was identified by this RNA-seq analysis and confirmed by RT-PCR. However their role in CLL biology is currently not clear and this will require additional studies to sequence novel transcripts in leukemic cells and to determine whether alternative exon usage alters the function of the expressed protein.

## Conclusion

The main strength of RNA sequencing data is that besides providing expression analysis it can be further mined for a number of other genetic abnormalities, including splicing alterations, fusion transcripts, alternate transcription start sites, point mutations, novel transcripts, fusion genes etc. that will provide novel insights in this leukemia. As there is variability of expression in primary leukemic specimens and occasionally between RNA-seq data and qRT-PCR, further confirmation of RNA-seq data is required to obtain accurate information. Novel DEG and spliced transcripts were identified that potentially have biological significance in this leukemia and are valuable leads for discovery of novel biomarkers and therapeutic targets in this disease.

## Additional files

Additional file 1: **Alignment statistic summary of all 15 samples signal-end reads mapped to UCSC H. sapiens reference genome (build hg19) using Tophat alignment program.** (DOCX 19 kb)
Additional file 2: **Number of transcripts and genes in B cells, U-CLL and M-CLL.** Pair wise scatter plot matrix. (DOCX 148 kb)
Additional file 3: **Differentially expressed genes in CLL relative to B cells based on FPKM analysis.** (XLSX 214 kb)
Additional file 4: **IPA functional annotation of differentially expressed genes in CLL specimens.** (DOCX 19 kb)
Additional file 5: **List of genes in each functional pathway from IPA analysis.** (XLSX 27 kb)
Additional file 6: **Differentially expressed genes in M- and U-CLL specimens.** (XLSX 78 kb)
Additional file 7: **List of alternative spliced genes from MATS analysis, comparing CLL and B cell data.** (XLSX 45 kb)
Additional file 8: **List of skipped exon events tested in CLL specimens with PCR analysis.** (PPTX 119 kb)

## Abbreviations

AS: Alternative splicing
CGH: Comparative genomic hybridization
CLL: Chronic lymphocytic leukemia
DEG: Differentially expressed gene
FPKM: Fragments per Kilobase of exon per million fragments mapped
IGVH: Immunoglobulin variable region heavy chain
M-CLL: IGVH mutated CLL
MATS: Multivariate Analysis of Transcript Splicing
SE: Skipped exon
U-CLL: IGVH non-mutated CLL
