# Supplementary material for: Gene expression and splicing alterations analyzed by high throughput RNA sequencing of chronic lymphocytic leukemia specimens
Source: BMC Cancer. 2015 Oct 16;15:714. doi: 10.1186/s12885-015-1708-9 (PMC4609092; doi:10.1186/s12885-015-1708-9)
Supplement: Additional file 1: — Alignment statistic summary of all 15 samples signal-end reads mapped to UCSC H. sapiens reference genome (build hg19) using Tophat alignment program. (DOCX 19 kb) [file 12885_2015_1708_MOESM1_ESM.docx]

Supplementary data 1: Alignment statistic summary of all 15 samples signal-end reads mapped to UCSC H. sapiens reference genome (build hg19)

using Tophat alignment program.

|  | Normal | | | | | VH non-mutated | | | | | | VH mutated | | | |
| --- | --- | --- | --- | --- | --- | --- | --- | --- | --- | --- | --- | --- | --- | --- | --- |
|  | B1 | B2 | B3 | B4 | B5 | CLL6 | CLL9 | CLL25 | CLL28 | CLL40 | CLL44 | CLL26 | CLL32 | CLL37 | CLL39 |
| Raw reads | 85,489,378 | 43,439,094 | 31,329,690 | 37,883,998 | 44,835,145 | 48,202,142 | 39,121,781 | 50,823,066 | 101,527,997 | 54,313,809 | 45,764,812 | 44,998,606 | 39,475,399 | 44,908,292 | 37,496,949 |
| Total Reads aligned | 102,723,382 | 51,445,631 | 38,243,698 | 47,026,445 | 54,208,442 | 70,589,647 | 46,190,143 | 66,665,170 | 119,463,969 | 65,685,506 | 57,637,451 | 55,678,044 | 49,741,751 | 56,254,595 | 44,727,052 |
| Reads QC failed | 0 | 0 | 0 | 0 | 0 | 0 | 0 | 0 | 0 | 0 | 0 | 0 | 0 | 0 | 0 |
| Optical/PCR duplicate | 0 | 0 | 0 | 0 | 0 | 0 | 0 | 0 | 0 | 0 | 0 | 0 | 0 | 0 | 0 |
| Non Primary Hits | 26,164,409 | 13,053,648 | 10,115,125 | 12,432,452 | 13,648,836 | 26,241,174 | 12,295,499 | 19,234,342 | 32,164,084 | 17,736,788 | 15,102,983 | 14,437,610 | 13,535,029 | 14,370,319 | 12,005,026 |
| Unmapped reads | 0 | 0 | 0 | 0 | 0 | 0 | 0 | 0 | 0 | 0 | 0 | 0 | 0 | 0 | 0 |
| Multiple mapped reads | 9,713,972 | 4,754,689 | 3,778,126 | 4,576,908 | 5,086,542 | 12,773,885 | 5,204,441 | 8,825,980 | 14,116,782 | 8,017,971 | 6,794,570 | 6,678,911 | 6,276,297 | 6,136,634 | 5,366,449 |
| Uniquely mapped | 66,845,001 | 33,637,294 | 24,350,447 | 30,017,085 | 35,473,064 | 31,574,588 | 28,690,203 | 38,604,848 | 73,183,103 | 39,930,747 | 35,739,898 | 34,561,523 | 29,930,425 | 35,747,642 | 27,355,577 |
| % Uniquely mapped | 78% | 77% | 78% | 79% | 79% | 66% | 73% | 76% | 72% | 74% | 78% | 77% | 76% | 80% | 73% |
| Read-1 | 0 | 0 | 0 | 0 | 0 | 0 | 0 | 0 | 0 | 0 | 0 | 0 | 0 | 0 | 0 |
| Read-2 | 0 | 0 | 0 | 0 | 0 | 0 | 0 | 0 | 0 | 0 | 0 | 0 | 0 | 0 | 0 |
| Reads map to '+' | 33,412,428 | 16,812,836 | 12,183,250 | 15,002,649 | 17,723,618 | 15,775,208 | 14,327,929 | 19,279,806 | 36,506,010 | 19,939,536 | 17,875,686 | 17,264,867 | 14,948,144 | 17,859,272 | 13,645,219 |
| Reads map to '-' | 33,432,573 | 16,824,458 | 12,167,197 | 15,014,436 | 17,749,446 | 15,799,380 | 14,362,274 | 19,325,042 | 36,677,093 | 19,991,211 | 17,864,212 | 17,296,656 | 14,982,281 | 17,888,370 | 13,710,358 |
| Non-splice reads | 61,857,343 | 31,084,870 | 22,485,011 | 27,647,740 | 32,601,222 | 27,926,139 | 25,669,193 | 34,220,758 | 65,849,453 | 35,924,882 | 31,733,451 | 30,996,576 | 26,828,857 | 31,775,273 | 24,653,277 |
| Splice reads | 4,987,658 | 2,552,424 | 1,865,436 | 2,369,345 | 2,871,842 | 3,648,449 | 3,021,010 | 4,384,090 | 7,333,650 | 4,005,865 | 4,006,447 | 3,564,947 | 3,101,568 | 3,972,369 | 2,702,300 |

Distribution of unique reads mapped to coding sequence exon (CDS_exon), 5’ and 3’ untranslated regions (5’ and 3’UTR_Exons), introns and intergenic regions.

|  | Numer of reads | | | | | | | | | | | | | | |
| --- | --- | --- | --- | --- | --- | --- | --- | --- | --- | --- | --- | --- | --- | --- | --- |
| Group feature | B1 | B2 | B3 | B4 | B5 | CLL6 | CLL9 | CLL25 | CLL28 | CLL40 | CLL44 | CLL26 | CLL32 | CLL37 | CLL39 |
| CDS_Exons | 27541997 | 13866819 | 10382066 | 12911469 | 15546920 | 16165915 | 14858358 | 20380486 | 36042894 | 19761328 | 19033749 | 17434197 | 15214172 | 19148058 | 13356931 |
| 5'UTR_Exons | 2086307 | 987108 | 747999 | 1112791 | 1313289 | 1030900 | 866281 | 1308119 | 2242233 | 1289503 | 1385403 | 1195357 | 973495 | 1285771 | 839981 |
| 3'UTR_Exons | 13370536 | 6980742 | 4956330 | 5499063 | 6326544 | 6559983 | 7100813 | 8485623 | 18002935 | 9549699 | 7906914 | 7789520 | 6985197 | 7723835 | 6883306 |
| Introns | 20263202 | 9913466 | 6974018 | 8917036 | 10516711 | 4946208 | 4319533 | 6061872 | 12291811 | 6895929 | 5473065 | 6009003 | 4966564 | 5697077 | 4487018 |
