# Supplementary material for: Gene expression and splicing alterations analyzed by high throughput RNA sequencing of chronic lymphocytic leukemia specimens
Source: BMC Cancer. 2015 Oct 16;15:714. doi: 10.1186/s12885-015-1708-9 (PMC4609092; doi:10.1186/s12885-015-1708-9)
Supplement: Additional file 2: — Number of transcripts and genes in B cells, U-CLL and M-CLL. Pair wise scatter plot matrix. (DOCX 148 kb) [file 12885_2015_1708_MOESM2_ESM.docx]

Supplementary data 2A: The number of transcript and genes detected in normal B cells, U-CLL and M-CLL using Cufflink assembly program.

|  | hg19 transcripts | Expressed | hg19 genes | Expressed |
| --- | --- | --- | --- | --- |
| Normal B cells | 44560 | 10396 | 23999 | 10081 |
| U-CLL | 44560 | 10494 | 23999 | 10111 |
| M-CLL | 44560 | 10402 | 23999 | 10068 |

Supplementary data 2B. Pair wise scatter plot matrix.

M-CLL specimen

U-CLL specimen

Normal B cells


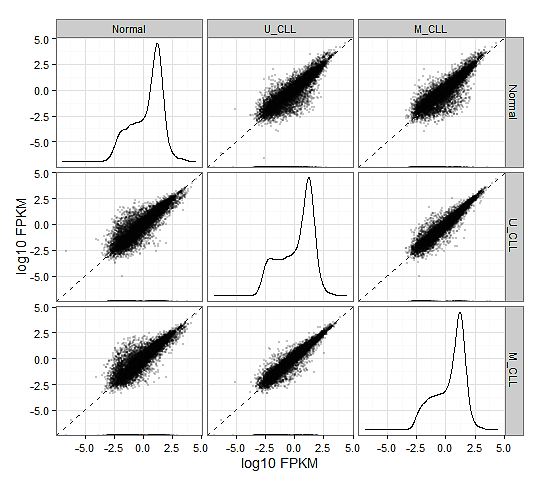


M-CLL specimen

U-CLL specimen

Normal B cells
