# Supplementary material for: Gene expression and splicing alterations analyzed by high throughput RNA sequencing of chronic lymphocytic leukemia specimens
Source: BMC Cancer. 2015 Oct 16;15:714. doi: 10.1186/s12885-015-1708-9 (PMC4609092; doi:10.1186/s12885-015-1708-9)
Supplement: Additional file 4: — IPA functional annotation of differentially expressed genes in CLL specimens. (DOCX 19 kb) [file 12885_2015_1708_MOESM4_ESM.docx]

Supplementary data 4: IPA functional annotation of DEG genes in CLL specimens. The vertical line indicates the cut-off significance of p<0.5. The list of genes in each Top 20 functional annotation shown, the list of genes in all the pathways is in Supplementary data 5.
