# Supplementary material for: Gene expression and splicing alterations analyzed by high throughput RNA sequencing of chronic lymphocytic leukemia specimens
Source: BMC Cancer. 2015 Oct 16;15:714. doi: 10.1186/s12885-015-1708-9 (PMC4609092; doi:10.1186/s12885-015-1708-9)
Supplement: Additional file 8: — List of skipped exon events tested in CLL specimens with PCR analysis. (PPTX 119 kb) [file 12885_2015_1708_MOESM8_ESM.pptx]

## Slide 1
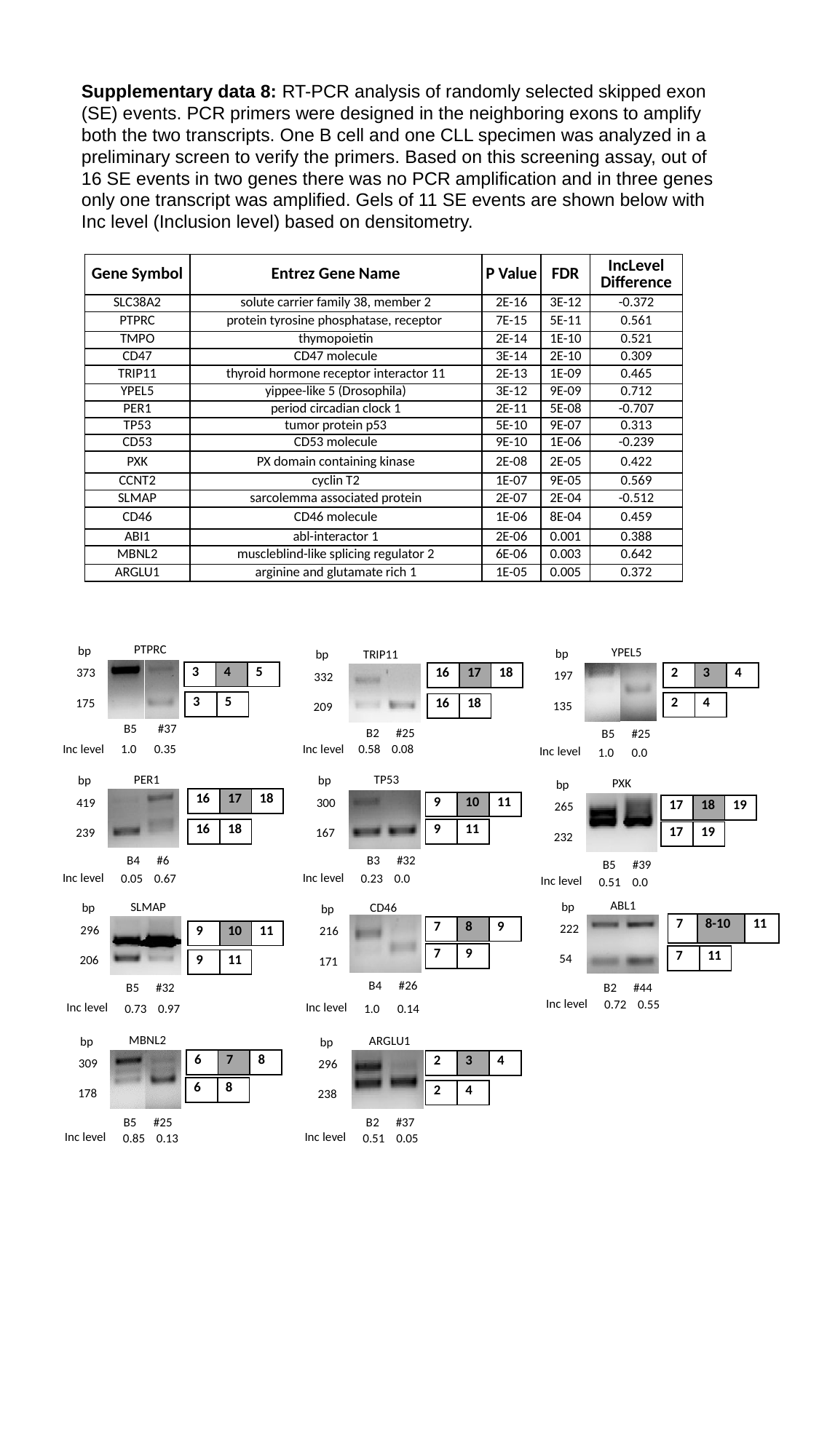

Supplementary data 8: RT-PCR analysis of randomly selected skipped exon (SE) events. PCR primers were designed in the neighboring exons to amplify both the two transcripts. One B cell and one CLL specimen was analyzed in a preliminary screen to verify the primers. Based on this screening assay, out of 16 SE events in two genes there was no PCR amplification and in three genes only one transcript was amplified. Gels of 11 SE events are shown below with Inc level (Inclusion level) based on densitometry.
| Gene Symbol | Entrez Gene Name | P Value | FDR | IncLevel Difference |
| --- | --- | --- | --- | --- |
| SLC38A2 | solute carrier family 38, member 2 | 2E-16 | 3E-12 | -0.372 |
| PTPRC | protein tyrosine phosphatase, receptor | 7E-15 | 5E-11 | 0.561 |
| TMPO | thymopoietin | 2E-14 | 1E-10 | 0.521 |
| CD47 | CD47 molecule | 3E-14 | 2E-10 | 0.309 |
| TRIP11 | thyroid hormone receptor interactor 11 | 2E-13 | 1E-09 | 0.465 |
| YPEL5 | yippee-like 5 (Drosophila) | 3E-12 | 9E-09 | 0.712 |
| PER1 | period circadian clock 1 | 2E-11 | 5E-08 | -0.707 |
| TP53 | tumor protein p53 | 5E-10 | 9E-07 | 0.313 |
| CD53 | CD53 molecule | 9E-10 | 1E-06 | -0.239 |
| PXK | PX domain containing kinase | 2E-08 | 2E-05 | 0.422 |
| CCNT2 | cyclin T2 | 1E-07 | 9E-05 | 0.569 |
| SLMAP | sarcolemma associated protein | 2E-07 | 2E-04 | -0.512 |
| CD46 | CD46 molecule | 1E-06 | 8E-04 | 0.459 |
| ABI1 | abl-interactor 1 | 2E-06 | 0.001 | 0.388 |
| MBNL2 | muscleblind-like splicing regulator 2 | 6E-06 | 0.003 | 0.642 |
| ARGLU1 | arginine and glutamate rich 1 | 1E-05 | 0.005 | 0.372 |
PTPRC
bp
373
175
1.0
0.35
Inc level
B5
#37
YPEL5
bp
197
135
1.0
0.0
Inc level
B5
#25
bp
TRIP11
332
209
0.58
0.08
Inc level
B2
#25
| 3 | 4 | 5 |
| --- | --- | --- |
| 16 | 17 | 18 |
| --- | --- | --- |
| 2 | 3 | 4 |
| --- | --- | --- |
| 3 | 5 |
| --- | --- |
| 2 | 4 |
| --- | --- |
| 16 | 18 |
| --- | --- |
PER1
bp
419
239
0.05
0.67
Inc level
B4
#6
TP53
bp
300
167
0.23
0.0
Inc level
B3
#32
PXK
bp
265
232
0.51
0.0
Inc level
B5
#39
| 16 | 17 | 18 |
| --- | --- | --- |
| 9 | 10 | 11 |
| --- | --- | --- |
| 17 | 18 | 19 |
| --- | --- | --- |
| 9 | 11 |
| --- | --- |
| 16 | 18 |
| --- | --- |
| 17 | 19 |
| --- | --- |
ABL1
bp
222
54
0.72
0.55
Inc level
B2
#44
SLMAP
bp
296
206
0.73
0.97
Inc level
B5
#32
CD46
bp
216
171
1.0
0.14
Inc level
B4
#26
| 7 | 8-10 | 11 |
| --- | --- | --- |
| 7 | 8 | 9 |
| --- | --- | --- |
| 9 | 10 | 11 |
| --- | --- | --- |
| 7 | 9 |
| --- | --- |
| 7 | 11 |
| --- | --- |
| 9 | 11 |
| --- | --- |
MBNL2
bp
309
178
0.85
0.13
Inc level
B5
#25
ARGLU1
bp
296
238
0.51
0.05
Inc level
B2
#37
| 6 | 7 | 8 |
| --- | --- | --- |
| 2 | 3 | 4 |
| --- | --- | --- |
| 6 | 8 |
| --- | --- |
| 2 | 4 |
| --- | --- |
